# Redetermination of 1-benzyl-3-furoyl-1-phenyl­thio­urea

**DOI:** 10.1107/S1600536808044085

**Published:** 2009-02-28

**Authors:** O. Estévez-Hernández, Rodrigo S. Corrêa, J. Ellena, J. Duque

**Affiliations:** a Laboratory of Molecular Engineering, Institute of Materials, University of Havana, Cuba; b Grupo de Cristalografía, Instituto de Física de São Carlos, Universidade de São Paulo, São Carlos, Brazil

## Abstract

The title compound, C_19_H_16_N_2_O_2_S, was synthesized from furoyl isothio­cyanate and *N*-benzyl­aniline in dry acetone and the structure redetermined. The structure [Otazo-Sánchez *et al.* (2001 ▶). *J. Chem. Soc. Perkin Trans. 2*, pp. 2211–2218] has been re-determined in order to establish the intramolecular and intermolecular inter­actions. The thio­urea group is in the thio­amide form. The thio­urea group makes a dihedral angle of 29.2 (6)° with the furoyl group. In the crystal structure, mol­ecules are linked by inter­molecular C—H⋯O inter­actions, forming one-dimensional chains along the *a* axis. An intra­molecular N—H⋯O hydrogen bond is also present.

## Related literature

For general background, see: Aly *et al.* (2007 ▶), Koch (2001 ▶), Estévez-Hernández *et al.* (2006 ▶). For related structures, see: Pérez *et al.* (2008 ▶). For the synthesis, see: Otazo-Sánchez *et al.* (2001 ▶).
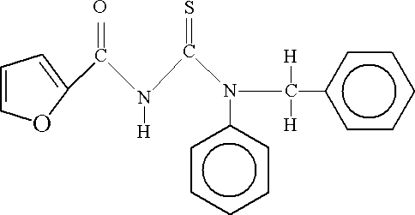


## Experimental

### 

#### Crystal data

C_19_H_16_N_2_O_2_S
*M* *_r_* = 336.41
Orthorhombic, 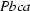

*a* = 12.7737 (3) Å
*b* = 8.8047 (2) Å
*c* = 31.2345 (7) Å
*V* = 3512.90 (14) Å^3^
*Z* = 8
Mo *K*α radiation
μ = 0.20 mm^−1^
*T* = 294 (2) K
0.54 × 0.22 × 0.19 mm

#### Data collection

Nonius KappaCCD diffractometer
Absorption correction: gaussian (Coppens *et al.*, 1965 ▶) *T*_min_ = 0.92, *T*_max_ = 0.971
19732 measured reflections
3536 independent reflections
2565 reflections with *I* > 2σ(*I*)
*R*_int_ = 0.058

#### Refinement

*R*[*F* ^2^ > 2σ(*F* ^2^)] = 0.041
*wR*(*F* ^2^) = 0.114
*S* = 1.03
3536 reflections
281 parameters
H atoms treated by a mixture of independent and constrained refinement
Δρ_max_ = 0.12 e Å^−3^
Δρ_min_ = −0.13 e Å^−3^

### 

Data collection: *COLLECT* (Nonius, 2000 ▶); cell refinement: *SCALEPACK* (Otwinowski & Minor, 1997 ▶); data reduction: *DENZO* (Otwinowski & Minor, 1997 ▶) and *SCALEPACK*; program(s) used to solve structure: *SHELXS97* (Sheldrick, 2008 ▶); program(s) used to refine structure: *SHELXL97* (Sheldrick, 2008 ▶); molecular graphics: *ORTEP-3 for Windows* (Farrugia, 1997 ▶) and *Mercury* (Bruno *et al.*, 2002 ▶); software used to prepare material for publication: *WinGX* (Farrugia, 1999 ▶).

## Supplementary Material

Crystal structure: contains datablocks global, I. DOI: 10.1107/S1600536808044085/bq2112sup1.cif

Structure factors: contains datablocks I. DOI: 10.1107/S1600536808044085/bq2112Isup2.hkl

Additional supplementary materials:  crystallographic information; 3D view; checkCIF report

## Figures and Tables

**Table 1:** Hydrogen-bond geometry (Å, °)

*D*—H⋯*A*	*D*—H	H⋯*A*	*D*⋯*A*	*D*—H⋯*A*
N1—H1⋯O2	0.84 (2)	2.25 (2)	2.677 (2)	111 (2)
C6—H6⋯O1^i^	0.92 (2)	2.40 (2)	3.315 (3)	172 (2)
C8—H8⋯O1^ii^	0.92 (2)	2.57 (2)	3.242 (2)	131 (2)

Symmetry codes: (i) 
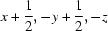
; (ii) 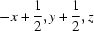.

**Fig. 1.**
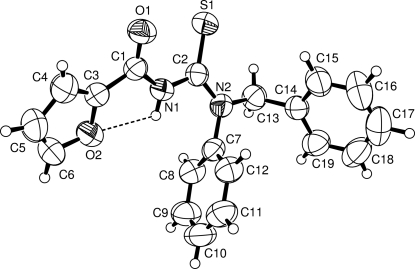
The molecular structure of the title compound. Displacement ellipsoids are drawn at the 50% probability level. The intramolecular N—H···O hydrogen bond is shown as a dashed line.

**Fig. 2.**
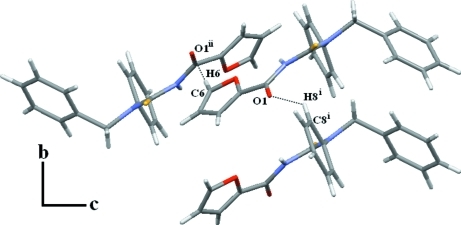
View of the crystal packing of the title compound. Intermolecular hydrogen bonds are shown as dashed lines. [Symmetry codes: (i) -x+1/2, y-1/2, z; (ii) x+1/2, -y+1/2, -z]

## supplementary crystallographic information

### Comment

The importance of aroylthioureas is largely in heterocyclic syntheses and many of these substrates have interesting biological activities. Aroylthioureas have also been found to have applications in metal complexes and molecular electronics (Aly *et al.*, 2007, Estévez-Hernández *et al.*, 2006). The structure of the title compound (I), Fig.1, has been redetermined and the result adds significantly to the information already in the public domain (Otazo-Sánchez *et al.*, 2001), especially about the intra and intermolecular interactions (not reported previously). The data and the refinement of the structure are also of better quality (present refinement: *R*: 0.0410 and *wR*: 0.1137; previous refinement: *R*: 0.1450 and *wR*: 0.2356). The main bond lengths and angles are within the ranges obtained for similar compounds (Koch *et al.*, 2001; Pérez *et al.*, 2008). The C2—S1 and C1—O1 bonds show typical double-bond character. However, the C—N bond lengths, C1—N1, C2—N1, C2—N2 are shorter than the normal C—N single-bond length of about 1.48 Å. These results can be explained by the existence of resonance in this part of the molecule. The central thiourea fragment (N1—C2—S1—N2) makes dihedral angle of 29,2(6)° with the furan carbonyl (O1—O2—C1—C3—C6) group, whereas the C7—C12 benzene ring is inclined by 84,7(6)°. The crystal structure is stabilized principally by the intramolecular N1—H···O2 hydrogen bond (Fig.1 and Table 1). In the crystal structure symmetry related molecules are linked by two different C—H···O1 interactions (C8—H···O1, 3.2<D<3.6 Å and θ>110°) and (C6—H···O1, 3.2<D<3.6 Å and θ>150°) to form one-dimensional chains along the *a*-axis (Fig. 2 and Table 1).

### Experimental

The title compound, (I), was synthesized according to a procedure described by Otazo-Sánchez *et al.* (2001), by converting furoyl chloride into furoyl isothiocyanate and then condensing with N-benzylaniline. The resulting solid product was crystallized from ethanol yielding X-ray quality single crystals (m.p. 127–128°C). Elemental analysis for C_19_H_16_N_2_O_2_S found: C 59.95, H 4.60, N 10.74, S 11.21%; calculated: C 60.00, H 4.62, N 10.76, S 11.31%.

### Refinement

All H atoms were refined with *U*_iso_(H)=1.2*U*_eq_(C/N).

### Crystal data

**Table:**

C_19_H_16_N_2_O_2_S	*F*(000) = 1408
*M**_r_* = 336.41	*D*_x_ = 1.272 Mg m^−^^3^
Orthorhombic, *P**b**c**a*	Mo *K*α radiation, λ = 0.71073 Å
Hall symbol: -P 2ac 2ab	Cell parameters from 19732 reflections
*a* = 12.7737 (3) Å	θ = 2.9–26.4°
*b* = 8.8047 (2) Å	µ = 0.20 mm^−^^1^
*c* = 31.2345 (7) Å	*T* = 294 K
*V* = 3512.90 (14) Å^3^	Prism, yellow
*Z* = 8	0.54 × 0.22 × 0.19 mm

### Data collection

**Table:**

Nonius KappaCCD diffractometer	2565 reflections with *I* > 2σ(*I*)
CCD rotation images, thick slices scans	*R*_int_ = 0.058
Absorption correction: gaussian (Coppens *et al.*, 1965)	θ_max_ = 26.4°, θ_min_ = 3.1°
*T*_min_ = 0.92, *T*_max_ = 0.971	*h* = −15→15
19732 measured reflections	*k* = −11→8
3536 independent reflections	*l* = −38→39

### Refinement

**Table:**

Refinement on *F*^2^	0 restraints
Least-squares matrix: full	H atoms treated by a mixture of independent and constrained refinement
*R*[*F*^2^ > 2σ(*F*^2^)] = 0.041	*w* = 1/[σ^2^(*F*_o_^2^) + (0.0583*P*)^2^ + 0.343*P*] where *P* = (*F*_o_^2^ + 2*F*_c_^2^)/3
*wR*(*F*^2^) = 0.114	(Δ/σ)_max_ < 0.001
*S* = 1.03	Δρ_max_ = 0.12 e Å^−^^3^
3536 reflections	Δρ_min_ = −0.13 e Å^−^^3^
281 parameters	

### Special details

**Table:**

Geometry. All e.s.d.'s (except the e.s.d. in the dihedral angle between two l.s. planes) are estimated using the full covariance matrix. The cell e.s.d.'s are taken into account individually in the estimation of e.s.d.'s in distances, angles and torsion angles; correlations between e.s.d.'s in cell parameters are only used when they are defined by crystal symmetry. An approximate (isotropic) treatment of cell e.s.d.'s is used for estimating e.s.d.'s involving l.s. planes.

### Fractional atomic coordinates and isotropic or equivalent isotropic displacement parameters (Å^2^)

**Table:**

	*x*	*y*	*z*	*U*_iso_*/*U*_eq_	
C1	0.26117 (12)	0.19869 (17)	0.05040 (4)	0.0577 (4)	
C2	0.26889 (12)	0.37199 (18)	0.11358 (4)	0.0577 (4)	
C3	0.32563 (12)	0.1570 (2)	0.01356 (5)	0.0619 (4)	
C4	0.31207 (19)	0.0530 (3)	−0.01689 (6)	0.0842 (6)	
C5	0.39938 (19)	0.0609 (3)	−0.04428 (7)	0.0952 (7)	
C6	0.46044 (18)	0.1672 (3)	−0.02896 (6)	0.0861 (6)	
C7	0.45449 (12)	0.39652 (19)	0.13227 (5)	0.0599 (4)	
C8	0.51367 (15)	0.5088 (2)	0.11332 (6)	0.0723 (5)	
C9	0.61918 (16)	0.4856 (3)	0.10720 (7)	0.0865 (6)	
C10	0.66461 (18)	0.3518 (3)	0.11944 (7)	0.0909 (6)	
C11	0.60580 (16)	0.2400 (3)	0.13808 (7)	0.0866 (6)	
C12	0.50023 (15)	0.2623 (2)	0.14502 (6)	0.0747 (5)	
C13	0.32176 (18)	0.5218 (2)	0.17699 (6)	0.0706 (4)	
C14	0.34629 (12)	0.44677 (18)	0.21911 (5)	0.0615 (4)	
C15	0.28076 (19)	0.3390 (3)	0.23609 (6)	0.0877 (6)	
C16	0.3022 (3)	0.2697 (3)	0.27438 (8)	0.1152 (9)	
C17	0.3882 (3)	0.3076 (3)	0.29671 (8)	0.1110 (9)	
C18	0.4556 (2)	0.4131 (4)	0.28073 (8)	0.1023 (8)	
C19	0.43495 (17)	0.4839 (3)	0.24164 (6)	0.0822 (5)	
N1	0.30881 (11)	0.30009 (16)	0.07753 (4)	0.0607 (4)	
N2	0.34480 (10)	0.42232 (15)	0.14007 (4)	0.0613 (3)	
O1	0.17529 (9)	0.14370 (14)	0.05539 (4)	0.0732 (3)	
O2	0.41816 (9)	0.23024 (15)	0.00713 (4)	0.0762 (3)	
S1	0.14167 (3)	0.39752 (6)	0.121403 (14)	0.07098 (18)	
H1	0.3720 (15)	0.319 (2)	0.0721 (6)	0.078 (6)*	
H4	0.2628 (18)	0.001 (3)	−0.0187 (6)	0.092 (7)*	
H5	0.4127 (17)	0.003 (3)	−0.0693 (7)	0.109 (7)*	
H6	0.5238 (18)	0.211 (3)	−0.0356 (7)	0.107 (7)*	
H8	0.4814 (14)	0.597 (2)	0.1052 (5)	0.075 (5)*	
H9	0.660 (2)	0.558 (3)	0.0925 (8)	0.120 (8)*	
H10	0.734 (2)	0.335 (3)	0.1144 (8)	0.133 (9)*	
H11	0.6356 (17)	0.143 (3)	0.1469 (7)	0.108 (7)*	
H12	0.4597 (16)	0.188 (2)	0.1588 (6)	0.088 (6)*	
H13A	0.3612 (15)	0.614 (2)	0.1742 (6)	0.081 (6)*	
H13B	0.2478 (17)	0.544 (2)	0.1764 (5)	0.082 (5)*	
H15	0.2202 (18)	0.315 (3)	0.2208 (7)	0.112 (8)*	
H16	0.255 (3)	0.181 (4)	0.2858 (10)	0.180 (12)*	
H17	0.404 (2)	0.260 (3)	0.3255 (9)	0.131 (8)*	
H18	0.510 (2)	0.441 (3)	0.2933 (8)	0.130 (10)*	
H19	0.4794 (17)	0.555 (3)	0.2293 (7)	0.103 (7)*	

### Atomic displacement parameters (Å^2^)

**Table:**

	*U*^11^	*U*^22^	*U*^33^	*U*^12^	*U*^13^	*U*^23^
C1	0.0526 (8)	0.0640 (9)	0.0563 (8)	0.0003 (7)	−0.0034 (6)	0.0060 (7)
C2	0.0570 (9)	0.0602 (9)	0.0560 (8)	0.0011 (7)	0.0010 (6)	0.0069 (7)
C3	0.0551 (8)	0.0738 (10)	0.0569 (8)	−0.0019 (8)	−0.0019 (7)	0.0037 (8)
C4	0.0826 (14)	0.0989 (16)	0.0711 (11)	−0.0108 (13)	−0.0020 (10)	−0.0153 (10)
C5	0.1014 (16)	0.1183 (18)	0.0659 (11)	0.0129 (14)	0.0064 (11)	−0.0178 (12)
C6	0.0735 (12)	0.1153 (17)	0.0695 (11)	0.0085 (12)	0.0163 (10)	0.0017 (11)
C7	0.0562 (9)	0.0675 (10)	0.0559 (8)	0.0011 (8)	−0.0065 (7)	−0.0052 (7)
C8	0.0659 (11)	0.0739 (12)	0.0772 (11)	0.0012 (9)	−0.0067 (8)	0.0021 (9)
C9	0.0644 (12)	0.0936 (15)	0.1013 (14)	−0.0087 (11)	0.0005 (10)	0.0047 (12)
C10	0.0557 (11)	0.1095 (18)	0.1075 (15)	0.0044 (12)	−0.0053 (10)	−0.0069 (13)
C11	0.0733 (13)	0.0878 (15)	0.0988 (14)	0.0177 (11)	−0.0119 (11)	−0.0013 (12)
C12	0.0697 (11)	0.0729 (12)	0.0814 (11)	0.0032 (10)	−0.0052 (9)	0.0025 (9)
C13	0.0747 (12)	0.0698 (12)	0.0674 (10)	0.0067 (10)	−0.0003 (9)	−0.0074 (8)
C14	0.0624 (9)	0.0622 (9)	0.0600 (8)	0.0016 (8)	0.0025 (7)	−0.0115 (7)
C15	0.0932 (14)	0.1008 (15)	0.0691 (11)	−0.0237 (12)	0.0073 (10)	−0.0063 (10)
C16	0.157 (3)	0.115 (2)	0.0739 (13)	−0.0213 (18)	0.0218 (16)	0.0066 (13)
C17	0.161 (3)	0.1054 (19)	0.0661 (13)	0.0328 (19)	0.0046 (15)	0.0021 (13)
C18	0.0984 (17)	0.126 (2)	0.0826 (14)	0.0257 (15)	−0.0288 (13)	−0.0254 (14)
C19	0.0754 (12)	0.0875 (13)	0.0838 (12)	−0.0046 (11)	−0.0083 (10)	−0.0092 (11)
N1	0.0489 (7)	0.0748 (9)	0.0584 (7)	−0.0047 (6)	0.0032 (6)	−0.0032 (6)
N2	0.0592 (8)	0.0697 (8)	0.0552 (7)	0.0047 (6)	−0.0013 (6)	−0.0031 (6)
O1	0.0583 (6)	0.0822 (8)	0.0791 (7)	−0.0113 (6)	0.0047 (5)	−0.0040 (6)
O2	0.0632 (7)	0.0934 (9)	0.0719 (7)	−0.0070 (6)	0.0097 (5)	−0.0042 (6)
S1	0.0554 (3)	0.0834 (3)	0.0741 (3)	0.0051 (2)	0.00711 (18)	−0.0001 (2)

### Geometric parameters (Å, °)

**Table:**

C1—O1	1.2092 (18)	C10—C11	1.368 (3)
C1—N1	1.3731 (19)	C10—H10	0.91 (3)
C1—C3	1.462 (2)	C11—C12	1.380 (3)
C2—N2	1.349 (2)	C11—H11	0.97 (2)
C2—N1	1.389 (2)	C12—H12	0.94 (2)
C2—S1	1.6586 (16)	C13—N2	1.478 (2)
C3—C4	1.332 (3)	C13—C14	1.505 (2)
C3—O2	1.361 (2)	C13—H13A	0.96 (2)
C4—C5	1.407 (3)	C13—H13B	0.97 (2)
C4—H4	0.78 (2)	C14—C15	1.372 (3)
C5—C6	1.309 (3)	C14—C19	1.373 (2)
C5—H5	0.95 (2)	C15—C16	1.370 (3)
C6—O2	1.368 (2)	C15—H15	0.93 (2)
C6—H6	0.92 (2)	C16—C17	1.344 (4)
C7—C12	1.377 (2)	C16—H16	1.05 (4)
C7—C8	1.378 (2)	C17—C18	1.361 (4)
C7—N2	1.440 (2)	C17—H17	1.01 (3)
C8—C9	1.376 (3)	C18—C19	1.396 (3)
C8—H8	0.917 (19)	C18—H18	0.84 (3)
C9—C10	1.368 (3)	C19—H19	0.93 (2)
C9—H9	0.94 (3)	N1—H1	0.841 (19)
			
O1—C1—N1	125.65 (14)	C7—C12—H12	119.7 (13)
O1—C1—C3	120.79 (14)	C11—C12—H12	120.8 (13)
N1—C1—C3	113.54 (13)	N2—C13—C14	112.36 (14)
N2—C2—N1	112.52 (14)	N2—C13—H13A	109.1 (11)
N2—C2—S1	124.70 (12)	C14—C13—H13A	110.0 (11)
N1—C2—S1	122.74 (12)	N2—C13—H13B	107.6 (11)
C4—C3—O2	109.47 (16)	C14—C13—H13B	108.1 (10)
C4—C3—C1	131.42 (17)	H13A—C13—H13B	109.7 (17)
O2—C3—C1	119.10 (14)	C15—C14—C19	118.04 (19)
C3—C4—C5	107.3 (2)	C15—C14—C13	120.96 (17)
C3—C4—H4	123.9 (16)	C19—C14—C13	121.00 (18)
C5—C4—H4	128.8 (16)	C16—C15—C14	121.6 (2)
C6—C5—C4	106.6 (2)	C16—C15—H15	120.8 (15)
C6—C5—H5	125.4 (14)	C14—C15—H15	117.6 (15)
C4—C5—H5	128.0 (14)	C17—C16—C15	120.4 (3)
C5—C6—O2	110.87 (19)	C17—C16—H16	118.4 (18)
C5—C6—H6	137.7 (14)	C15—C16—H16	121.1 (19)
O2—C6—H6	111.4 (14)	C16—C17—C18	119.8 (2)
C12—C7—C8	120.46 (17)	C16—C17—H17	121.5 (15)
C12—C7—N2	119.95 (15)	C18—C17—H17	118.7 (15)
C8—C7—N2	119.55 (15)	C17—C18—C19	120.4 (2)
C9—C8—C7	119.4 (2)	C17—C18—H18	123.7 (19)
C9—C8—H8	121.8 (12)	C19—C18—H18	115.9 (19)
C7—C8—H8	118.8 (11)	C14—C19—C18	119.8 (2)
C10—C9—C8	120.3 (2)	C14—C19—H19	117.0 (13)
C10—C9—H9	119.0 (15)	C18—C19—H19	123.2 (13)
C8—C9—H9	120.6 (15)	C1—N1—C2	129.35 (14)
C11—C10—C9	120.4 (2)	C1—N1—H1	115.3 (12)
C11—C10—H10	119.4 (17)	C2—N1—H1	115.3 (12)
C9—C10—H10	120.2 (17)	C2—N2—C7	122.93 (13)
C10—C11—C12	120.0 (2)	C2—N2—C13	122.01 (14)
C10—C11—H11	122.4 (13)	C7—N2—C13	114.78 (14)
C12—C11—H11	117.6 (13)	C3—O2—C6	105.79 (15)
C7—C12—C11	119.5 (2)		
			
O1—C1—C3—C4	−6.5 (3)	C16—C17—C18—C19	1.1 (4)
N1—C1—C3—C4	172.34 (19)	C15—C14—C19—C18	−0.4 (3)
O1—C1—C3—O2	174.73 (14)	C13—C14—C19—C18	179.69 (18)
N1—C1—C3—O2	−6.5 (2)	C17—C18—C19—C14	−0.1 (3)
O2—C3—C4—C5	−0.6 (2)	O1—C1—N1—C2	−6.2 (3)
C1—C3—C4—C5	−179.47 (18)	C3—C1—N1—C2	175.04 (15)
C3—C4—C5—C6	0.4 (3)	N2—C2—N1—C1	159.13 (15)
C4—C5—C6—O2	−0.1 (3)	S1—C2—N1—C1	−23.1 (2)
C12—C7—C8—C9	0.0 (3)	N1—C2—N2—C7	−2.9 (2)
N2—C7—C8—C9	177.88 (16)	S1—C2—N2—C7	179.43 (12)
C7—C8—C9—C10	0.6 (3)	N1—C2—N2—C13	170.71 (14)
C8—C9—C10—C11	−0.3 (3)	S1—C2—N2—C13	−7.0 (2)
C9—C10—C11—C12	−0.6 (3)	C12—C7—N2—C2	−84.39 (19)
C8—C7—C12—C11	−1.0 (3)	C8—C7—N2—C2	97.73 (19)
N2—C7—C12—C11	−178.84 (16)	C12—C7—N2—C13	101.60 (18)
C10—C11—C12—C7	1.3 (3)	C8—C7—N2—C13	−76.27 (18)
N2—C13—C14—C15	−76.7 (2)	C14—C13—N2—C2	115.66 (17)
N2—C13—C14—C19	103.2 (2)	C14—C13—N2—C7	−70.3 (2)
C19—C14—C15—C16	0.0 (3)	C4—C3—O2—C6	0.5 (2)
C13—C14—C15—C16	179.9 (2)	C1—C3—O2—C6	179.59 (15)
C14—C15—C16—C17	0.9 (4)	C5—C6—O2—C3	−0.3 (2)
C15—C16—C17—C18	−1.4 (4)		

### Hydrogen-bond geometry (Å, °)

**Table:**

*D*—H···*A*	*D*—H	H···*A*	*D*···*A*	*D*—H···*A*
N1—H1···O2	0.84 (2)	2.25 (2)	2.677 (2)	111 (2)
C6—H6···O1^i^	0.92 (2)	2.40 (2)	3.315 (3)	172 (2)
C8—H8···O1^ii^	0.92 (2)	2.57 (2)	3.242 (2)	131 (2)

Symmetry codes: (i) *x*+1/2, −*y*+1/2, −*z*; (ii) −*x*+1/2, *y*+1/2, *z*.

## References

[bb1] Aly, A. A., Ahmed, E. K., El-Mokadem, K. M. & Hegazy, M. E. F. (2007). *J. Sulfur Chem.* **28**, 73–93.

[bb2] Bruno, I. J., Cole, J. C., Edgington, P. R., Kessler, M., Macrae, C. F., McCabe, P., Pearson, J. & Taylor, R. (2002). *Acta Cryst.* B**58**, 389–397. 10.1107/s0108768102003324 12037360

[bb3] Coppens, P., Leiserowitz, L. & Rabinovich, D. (1965). *Acta Cryst.* **18**, 1035–1038.

[bb4] Estévez-Hernández, O., Naranjo-Rodríguez, I., Hidalgo-Hidalgo de Cisneros, J. L. & Reguera, E. (2006). *Spectrochim. Acta Part A*, **64**, 961–971. 10.1016/j.saa.2005.09.005 16330247

[bb5] Farrugia, L. J. (1997). *J. Appl. Cryst.* **30**, 565.

[bb6] Farrugia, L. J. (1999). *J. Appl. Cryst.* **32**, 837–838.

[bb7] Koch, K. R. (2001). *Coord. Chem. Rev.* **216**–**217**, 473–488.

[bb8] Nonius (2000). *COLLECT* Nonius BV, Delft, The Netherlands.

[bb9] Otazo-Sánchez, E., Pérez-Marín, L., Estévez-Hernández, O., Rojas-Lima, S. & Alonso-Chamorro, J. (2001). *J. Chem. Soc. Perkin Trans. 2*, pp. 2211–2218.

[bb10] Otwinowski, Z. & Minor, W. (1997). *Methods in Enzymology*, Vol. 276, Macromolecular Crystallography, Part A, edited by C. W. Carter Jr & R. M. Sweet, pp. 307–326. New York: Academic Press.

[bb11] Pérez, H., Mascarenhas, Y., Estévez-Hernández, O., Santos, S. Jr & Duque, J. (2008). *Acta Cryst.* E**64**, o513. 10.1107/S1600536807068687 PMC2960311 21201532

[bb12] Sheldrick, G. M. (2008). *Acta Cryst.* A**64**, 112–122. 10.1107/S0108767307043930 18156677

